# Inter-subject Registration of Functional Images: Do We Need Anatomical Images?

**DOI:** 10.3389/fnins.2018.00064

**Published:** 2018-02-14

**Authors:** Elvis Dohmatob, Gael Varoquaux, Bertrand Thirion

**Affiliations:** Parietal Team, INRIA, CEA, Université Paris-Saclay, Gif-sur-Yvette, France

**Keywords:** functional MRI, human brain-mapping, high-resolution EPI, inter-subject registration, B0 inhomogeneity, distortions

## Abstract

In Echo-Planar Imaging (EPI)-based Magnetic Resonance Imaging (MRI), inter-subject registration typically uses the subject's T1-weighted (T1w) anatomical image to learn deformations of the subject's brain onto a template. The estimated deformation fields are then applied to the subject's EPI scans (functional or diffusion-weighted images) to warp the latter to a template space. Historically, such indirect T1w-based registration was motivated by the lack of clear anatomical details in low-resolution EPI images: a direct registration of the EPI scans to template space would be futile. A central prerequisite in such indirect methods is that the anatomical (aka the T1w) image of each subject is well aligned with their EPI images via rigid coregistration. We provide experimental evidence that things have changed: nowadays, there is a decent amount of anatomical contrast in high-resolution EPI data. That notwithstanding, EPI distortions due to B0 inhomogeneities cannot be fully corrected. Residual uncorrected distortions induce non-rigid deformations between the EPI scans and the same subject's anatomical scan. In this manuscript, we contribute a computationally cheap pipeline that leverages the high spatial resolution of modern EPI scans for direct inter-subject matching. Our pipeline is direct and does not rely on the T1w scan to estimate the inter-subject deformation. Results on a large dataset show that this new pipeline outperforms the classical indirect T1w-based registration scheme, across a variety of post-registration quality-assessment metrics including: Normalized Mutual Information, relative variance (variance-to-mean ratio), and to a lesser extent, improved peaks of group-level General Linear Model (GLM) activation maps.

## 1. Introduction

Registering brain images from different subjects in a common space (for example, the MNI space Collins et al., 1994; Mazziotta et al., 1995), is an essential step in any multi-subject analysis pipeline (Friston et al., 1995). Indeed, a voxel-to-voxel correspondence across subjects is needed for group-level statistics on brain maps to make sense. In addition, the use of a standard space opens the possibility to share results in a consistent fashion, hence the comparison of experiments and meta-analysis (Wager et al., 2007; Gorgolewski et al., 2015). This is especially true in functional Magnetic Resonance Imaging (fMRI) studies in which the activations might span just a few voxels in diameter.

Traditionally, a pipeline for registering functional images proceeds as follows. EPI and T1w images are rigidly aligned in a primary step called *coregistration*; then one applies the T1w → template transformation—estimated in a separate step—to the EPI images to warp them from subject to template space. As regards coregistration, it is assumed that the T1w and EPI images of the same subject could be properly aligned to one another via a rigid (affine) transformation. Thus, one typically assumes that distortion correction is good enough so that the EPI can be realigned to the T1w image with a rigid transformation. Historically, such an indirect T1w-based method for preprocessing functional images has been prompted by the fact that learning a deformation from the subject's T1w image to a template is easier, due to the relatively high anatomical contrast in T1w images, than learning a deformation from the subject's EPI image to the template.

**Figure 1:**
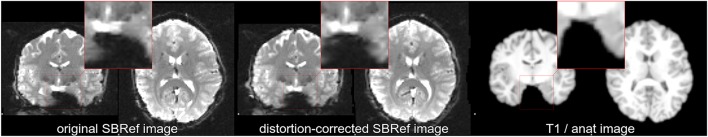
Non-linear mismatch between EPI and T1w image of the same subject of the HCP dataset (Van Essen et al., 2012), before and after distortion-correction. **(Left)** Single-band high-resolution EPI (SBRef) image of the same subject. Notice the large distortions along the Left-Right direction (inside the highlighted patches). **(Center)** Distortion-corrected single-band EPI image. Here, the distortion-correction managed to undo most—but not all—of the distortions. Even after distortion correction, there are minor shape differences between the EPI and the T1 image of the subject **(Right)**. The same native-space coordinates where used in all of the three plots.

However, it is widely known that even after correction efforts, high-resolution (3 T and above) EPI sequences suffer residual from distortions that push them non-linearly out-of-match relative to the T1w image of the same subject (see Renvall et al., 2016 for example), and so the two cannot be geometrically matched by a simple coregistration step. As illustrated in Figure 1, this is the case, for example, with the Human Connectome Project (HCP) (Van Essen et al., 2012) dataset, a reference dataset that contains high-quality EPI data acquired using state-of-the-art sequences, yet with severe distortions (Wan et al., 1997; Mangin et al., 2002; Zeng and Constable, 2002; Andersson et al., 2003). Thus, EPI-to-anatomy registration is nowadays typically treated as a non-linear registration problem (e.g., using boundary-based registration—BBR), after EPI distortion correction. Indeed, as discussed in the literature (e.g., Freire et al., 2002), EPI distortions and signal loss related to B0 inhomogeneities cannot be separated with registration based techniques, that are compensatory operations. Consequently, the quest for efficient combined distortion correction and coregistration method is still largely an open question.

In the present contribution, we formally test whether replacing the anatomical image by a high-quality EPI image works as well. For this we use a large dataset, obtained from the HCP dataset (Van Essen et al., 2012), and comprising 110 subjects. We find that the direct EPI-to-EPI registration pipeline yields higher inter-subject similarity: in particular, it maps more accurately raw BOLD contrasts and image outline, as measured by standard evaluation metrics. The impact on the results of group level analyses (from task and resting-state functional MRI) is however minor, meaning that between-subject variability is not primarily dominated by geometric aspects. This last point is discussed in detail in section 6. One should mention that some ultra-modern acquisition techniques and hardware setup make it possible to obtain very fine anatomical details in sub-millimeter EPI images at high field (7T) (Heidemann et al., 2012; Renvall et al., 2016). The concern of our paper is to investigate whether one can still bypass the anatomical scan and instead use an EPI image of worse resolution, say 2 mm^3^ as in the HCP functional data (Van Essen et al., 2012).

## 2. Materials and methods

### 2.1. An important note on normalization

Let us begin by stressing that the *normalization* problem (i.e., registration to a standard template) is not addressed in our work. We concentrate on inter-subject (non-linear) *registration*, since our goal is to show the benefits of using EPI images in place of anatomical images in pipelines. We also note that there is an increasing concern in the literature that in the future, normalization will be based on techniques using more fine-grained information like multi-modal atlases (tissue probability maps, functional parcellation maps, etc.) (Amunts et al., 2014), and anatomical maps (Waehnert et al., 2016), which link directly the functional competence of cortical areas directly with myeloarchitecture, and thence to cyto-architecture (Turner, 2016).

### 2.2. General preprocessing procedures

#### 2.2.1. Motion correction

During acquisitions, participant's head moves in the scanner, at least due to respiratory motion. This head movement induces an approximately rigid mismatch between different volumes acquired in the same run. Motion correction is done to remove this source of intra-subject variability. We used FSL's *flirt* tool (Smith et al., 2004) for motion correction.

#### 2.2.2. Distortion correction

Due to inhomogeneities in the ambient B0 field, the EPI images are distorted along the *phase-encoding direction* [Left-Right/Right-Left in the case of HCP dataset (Van Essen et al., 2012)]. See Figure 1. In our experiments, distortion correction (Jezzard and Balaban, 1995; Wan et al., 1997; Mangin et al., 2002; Zeng and Constable, 2002; Andersson et al., 2003) was achieved using the methods described in Van Essen et al. (2012). Both methods use FSL's *topup* tool (Andersson et al., 2003; Smith et al., 2004) to estimate the deformation field due to B0 inhomogeneities (Glasser et al., 2013). Given the spin echo field maps for the LR (left-right) and RL (right-left) phase-encoding directions, the topup tool (Andersson et al., 2003; Smith et al., 2004) estimates the deformation field that when applied to the two volumes will maximize the similarity of the unwarped volumes. The similarity is gauged by the sum-of-squared differences between the unwarped images. This similarity metric is optimized via a Gauss-Newton algorithm for jointly finding the field and any movement that may have occurred between the two acquisitions. These transformation are composed with subsequently estimated transformations (warp-fields for registration to template, etc.) and applied to the 4D EPI images.

#### 2.2.3. Deformation model

We used ANTs' *Symmetric Normalization* (aka *SyN*) deformation model (Avants et al., 2008, 2011), that has been shown to be a state-of-the art method for non-linear registration (Klein et al., 2009). As done usually, we initialize a non-linear registration algorithm with a rigid-body registration algorithm. The former is simply meant to estimate an alignment for the bounding boxes of the images (thus ensuring a sufficiently large region of overlap). Concretely, we stack a 2-level pyramidal^1^ rigid transformation model (as initialization) with a 3-level pyramidal SyN deformation model. *Mattes mutual information* (Mattes et al., 2003) is used as the loss function.

This model is triggered in ANTs (Avants et al., 2008, 2011) using the following command-line options:


      --transform Affine[ 2.0 ]
      --metric Mattes[ ${fixed_img}.nii.gz, ${moving_img}.nii.gz, \
                1, 32, Random, 0.05 ]
      --convergence [ 1500x200, 1e-08, 20 ] --smoothing-sigmas \
                1.0x0.0vox
      --shrink-factors 2x1 --use-estimate-learning-rate-once 1
      --use-histogram-matching 1


### 2.3. The pipelines

**Figure 2:**
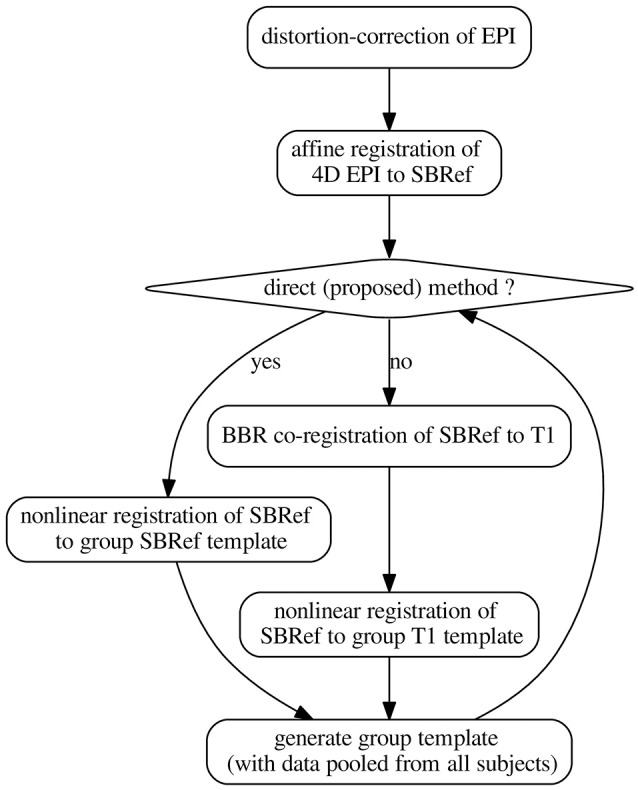
The pipelines. The template-generation step is done using ANTs (Avants et al., 2008, 2011). It pools registered data from all subjects. N.B.: SBRef, single-band reference image; i.e., high-resolution 3D volume EPI. As in Glasser et al. (2013), all the transformations are postponed and the original 4D EPI is resampled at the end by applying the composition of these transformations in a single step.

We now present constructions for the pipelines whose benchmark is the core of this work. The pipelines are schematized in Figure 2. All pipelines presented here were scripted in using command-line tools from FSL version 5.0 (Smith et al., 2004) for rigid registration, distortion correction, motion correction, ANTs (Avants et al., 2009) antsRegistration, antsApplyTranforms, and some custom scripts (for distortion correction) from the HCP scripts described in Glasser et al. (2013), hosted on Github. Except stated otherwise, all rigid registrations (motion correction, coregistration) were performed using FSL's *flirt* tool (Smith et al., 2004) with Normalized Mutual Information as cost the function (option: *-cost normmi*).

#### 2.3.1. Classical indirect T1w-based method

The classical indirect T1w-based pipeline for registration of EPI images can be schematized as follows^2^:

(1) $$\begin{array}{r} \begin{array}{c} \text{EPI}\to \text{templ}.=\underset{\text{non-linear}}{\underset{︸}{\left(\text{T}1\text{w}\to \text{templ}.\right)}}◦\underset{\text{linear}}{\underset{︸}{\left({\text{EPI}}_{0}\overset{\text{BBR}}{\to }\text{T}1\text{w}\right)}}◦\text{DistCorr} \end{array} \end{array}$$

in which a deformation of the subject's T1w image to a template is estimated and then applied to warp the same subject's EPI data. Here, EPI_0_ is any single-volume EPI image previously-coregistered with the 4D EPI sequence. Typical choices include: the middle volume of the EPI 4D time-series or the mean volume after motion correction. In our implementations, we used the former.

For the template, a subject is chosen and his/her T1w image is used as the template. For each other subject, (a) distortion correction is used to learn a non-linear undistorting warpfield, in a procedure already described in subsection 2.2 above. Then, (b) motion correction is done to realign the subject's EPI data to the mean thereof. The subject's T1w image is then aligned to this mean EPI image via coregistration (a rigid-body transformation). We use BBR (Greve and Fischl, 2009) for this coregistration step, for optimal results and fair comparison. BBR is a state-of-the-art functional-to-structural registration method driven by intensity difference across boundary (samples). It uses white-matter boundaries (via T1w segmentation). BBR needs good structural images (with little contrast bias), and some anatomical contrast in the EPI image, which is the case of the single-band high-resolution reference images in the HCP dataset (Van Essen et al., 2012). The implementation we use is based on the *epi_reg* command of FSL (Smith et al., 2004). However, since BBR is an affine correction method, it still suffers from the limitations explained in the introductory section. In particular, it is not resilient to distortions in the input EPI image. (c) ANTs is used to learn a deformation of the T1w image to the template. This produces a warped version of the T1w image, together with the corresponding deformation (and its inverse), for passing from the subject's space to the template space. Finally, (d) the deformations above—including all the postponed warpfields and affine transformations—are then applied to all EPI data that were previously coregistered to the T1w image of the subject. These EPI images include EPI images acquired on the same subject during another task, for instance. This one-step resampling procedure (see subsection 2.2) then produces a registered, motion-corrected, undistorted version of the input EPI data.

Then mean of all the registered T1w images is computed, and becomes the template henceforth. This procedure is iterated a couple of times.

#### 2.3.2. Our proposed *Direct* EPI-based non-linear inter-subject registration method

Our proposed pipeline operates just as the classical indirect T1w-based pipeline described above in section 2.3.1, except that the anatomical image is replaced with the single-band high-resolution EPI (the SBRef) image, which has more tissue contrast than any volume of the 4D EPI time-series being registered (Glasser et al., 2013), and also does not suffer from multi-band artifacts. The anatomical image is not used anywhere in this pipeline. The pipeline can be schematized as follows:

(2) $$\begin{array}{r} \begin{array}{c} \text{EPI}\to \text{templ}.=\underset{\text{non-linear}}{\underset{︸}{\left({\text{EPI}}_{0}\to \text{templ}.\right)}}◦\text{DistCorr}, \end{array} \end{array}$$

where we take EPI_0_ = Single-band high-resolution (SBRef) EPI image.

##### 2.3.2.1. A note on image interpolation (resampling)

To avoid degrading the images as they travel through a pipeline, we stack all intermediate transformations and postpone the resampling operations to the end of the pipeline. The transformations are then composed, and applied to the input image in a one-step resampling procedure based on the *ApplyTransforms* tool of the ANTs software (Avants et al., 2008, 2009). For example, affine transformations estimated during the motion correction step are converted to warpfields using FSL's *convertwarp* tool (Smith et al., 2004). FSL's *applywarp* tool (Smith et al., 2004) is then used to jointly apply this affine transformation warpfields and the warpfields corresponding to the deformations estimated by *topup* (Smith et al., 2004), that are stacked with subsequent transformations. We use this strategy in both pipelines.

## 3. Relation to previous works

### 3.1. Direct EPI-to-EPI non-linear inter-subject registration

The idea of EPI-to-EPI registration has already been used in the literature. Renvall et al. (2016) proposed a novel method to synthesize segmentable T1 anatomical contrast from high-resolution (7T) EPI functional images, in a way that alleviates geometric distortions between estimated functional activation patterns and the subject's underlying anatomy. Grabner et al. (2014) used high-resolution EPI (1.1 mm isotropic) data for different subjects acquired at 7 T to iteratively build a sequence of EPI-based study-specific templates of increasing quality/resolution (Grabner et al., 2006). The finest of these templates shows a great deal of anatomical detail. Group-level activation patterns for a finger-tapping task were also shown to be very accurately localized on the posterior bank of the central sulcus. The authors concluded that high-resolution (7T) EPI images contain enough anatomical information for inter-subject registration, and so one can effectively by-pass the anatomical image of subjects in the processing pipeline. This would for example allow one to avoid the classical coregistration step used to align the subject's EPI images to their anatomy. These works (Huang et al., 2010; Grabner et al., 2014) demonstrated results on small sample sizes, namely *n* = 16 and *n* = 10, respectively, and very high resolution (for functional images): 1.1 and 1 mm isotropic, respectively. In contrast, our results are on a much larger population—*n* = 110 subjects—and the data have lower spatial resolution, namely 2 mm isotropic. Moreover, we consider the EPI distortion problem (ignored by the cited works), which can greatly mar the results of registration procedures.

Our experiments confirm and extend the findings of (Huang et al., 2010; Grabner et al., 2014), but at an even lower resolution: 2 mm resolution, obtained from 3 T MRI, and on a much larger dataset. Using a variety of different task contrasts, we show that registration with our pipeline increases the pairwise Normalized Mutual Information (NMI) of subjects, over the classical pipeline; crucially, this leads to a decrease in residual post-registration inter-subject misalignement.

In comparison to Grabner et al. (2014), the pipeline we propose (refer to section 2.3.2) is much lighter computationally as it bypasses the potentially expensive and challenging step of generating a good template from EPI data (Grabner et al., 2006). Of course, this economy is more of a compromise between complexity and accuracy, and might be potential limitation of our contribution.

When preparing this manuscript, a recent work (Calhoun et al., 2017) was brought to our attention. This work is quite similar in spirit to ours and the conclusions reached are also similar. The work proposes to bypass the T1w scan during normalization of EPI images, in the presence of EPI distortions, and shows that EPI-based methods is significantly better than the traditional traditional T1w-based approach (is no distortions-correction is done) for more common lower resolution EPIs from multiple datasets as in the Autism Brain Imaging Data Exchange (ABIDE) (Di Martino et al., 2014).

### 3.2. Non-linear EPI-to-structural coregistration

A recent work (Wang et al., 2017) has considered the possibility of replacing the classical rigid EPI-to-structural coregistration step with a non-linear counterpart, and then running a non-linear structural-to-template registration as usual. They show that their method outperforms the method based on distortion correction and linear EPI-to-structural coregistration followed by structural-to-template registration as usual (see section 2.3.1). In contrast, our proposed method (refer to section 2.3.2) does not use the structural image at all.

## 4. Experiments

We now describe benchmarks done to compare the pipelines presented in this paper (subsection 2.3) on the task fMRI data of 110 subjects from the HCP dataset (Van Essen et al., 2012). The task fMRI data were acquired in an attempt to assess major domains that sample the diversity of neural systems, including: (1) visual, motion, somatosensory, and motor systems; (2) language processing (semantic and phonological processing); (3) social cognition (Theory of Mind); and (4) emotion processing. Due to time constraints, our benchmarks were run only on these 4 (out of a total of 7) tasks (i.e., protocols). Also, only data for LR (left-right) phase-encoding direction (Chang and Fitzpatrick, 1992) runs were used. In all the non-T1w-based pipelines, the single-band high-resolution (SBRef) image of the motor task was used to learn deformations of the subject's brain into template space (a fixed subject of the same dataset).

The estimated deformations were then applied to warp EPI data (previously coregistered to same subject's motor SBRef) acquired on the same subject during different task conditions, into template space. General Linear Models (GLMs) (Friston et al., 1994) were run using *nipy* (Gorgolewski et al., 2011), an open-source Python library for analysis of neuro-imaging data. For the purpose of reporting the results, the resulting maps were was co-registered to MNI space a posteriori.

### 4.1. Evaluation metrics

The pipelines were evaluated using the following qualitative and quantitative metrics.

#### 4.1.1. Normalized mutual information evaluation (NMI)

NMI is a popular similarity metric used to assess the quality of registration between two images, i.e., how well the images are aligned to one another (for example Maes et al., 1997). It is also the loss function minimized by many optimization algorithms in image registration. Formally, the NMI between two images *X*_1_ ~ *p*_*X*_1__ and *X*_2_ ~ *p*_*X*_2__ is defined by

(3) $$\begin{array}{r} NMI\left(X_{1},X_{2}\right)=\frac{I\left(X_{1},X_{2}\right)}{\sqrt{H\left(X_{1}\right)H\left(X_{2}\right)}}, \end{array}$$

where $H\left(X_{i}\right):=H\left(p_{X_{i}}\right):=\underset{x}{\sum }p_{X_{i}}\left(x\right)\log\left(p_{X_{i}}\left(x\right)\right)$ are the marginal entropies of images and *I*(*X*_1_, *X*_2_): = *H*(*X*_1_)−*H*(*X*_2_|*X*_1_) the mutual information between the voxel values.

A detailed overview of the use of the NMI metric in medical image registration can be found in Pluim et al. (2003). In our experiments, FSL's *flirt*^3^ tool (Smith et al., 2004) was used to compute NMI from subjects EPI' data after registration in both scenarios: the classical T1w-based method and our proposed direct EPI-to-EPI method.

#### 4.1.2. Inter-subject residual variance

In a good registration method, the residual subject-to-subject variance of the EPI image should be reduced. The aim of inter-subject registration is indeed to put subjects into spatial correspondence to facilitate later group analysis. To measure the quality of the different registration methods in this regards, we computed the relative variance—also known as variance-to-mean ratio (VMR)—across the different subjects after registration. This is defined for each voxel by

(4) $$\begin{array}{r} \text{VMR}=\frac{\text{variance image across subjects}}{\text{mean image across subjects}}. \end{array}$$

This produces a 3D statistic image in which regions of the brain that are not well registered across subjects are outlined.

#### 4.1.3. Group-level statistics and functional brain network patterns

Finally, in a successful inter-subject registration procedure, we expect the functional activation patterns to be more localized in space and to have higher peaks. Or could this effect could be masked by inter-subject variability in activation magnitude? This will be discussed in detail in the discussion section 6.

### 4.2. How many (plausible) pipelines are there?

It is worth noting that potentially hundreds of pipelines could have been considered for testing: should we do distortion correction? And if yes, how? Should we use linear or non-linear model for the deformation field? What degree should we use for the interpolating splines? In fact as noted in Poldrack et al. (2017), there are exponentially many pipelines that can be considered, based on the answers to the above choices. Of course some of these parameters have rule-of-thumb default values (for example, there is no doubt distortion correction is a good thing to do), but others are open to preferential choice. Thus, our goal is not to consider all possible pipelines, but to look at a more focal question: does direct EPI-based inter-registration outperform the traditional indirect T1w-based pipeline?

## 5. Results

We now present results of experiments performed on the task fMRI protocols of the HCP dataset (Van Essen et al., 2012). Refer to section 4 for detailed information about the experiments that we did. The different pipelines discussed in section 2.3 were used to register the data (inter-subject registration), and the quality of the registration was benchmarked using the different evaluation metrics discussed in section 4.

### 5.1. Normalized mutual information (NMI)

**Figure 3:**
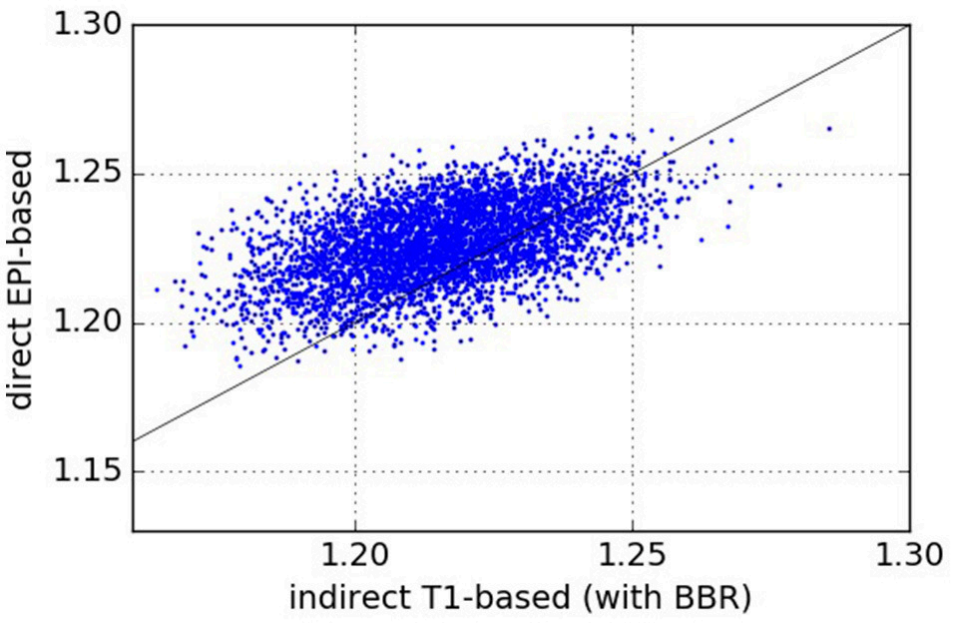
Normalized Mutual Information—NMI (higher values are better). Each point (*x, y*) on the plots such that *x* is the NMI of a given pair of subjects registered using the pipeline on the abscissa and *y* is the NMI of the same pair of subjects registered using the pipeline on the ordinate. From the one-sided We see that our proposed direct EPI-based pipeline significantly outperforms the classical indirect T1-based pipeline.

The results comparing across-subject NMI for the pipelines are presented in Figure 3. We see that NMI is in most cases higher through our approach, which means that our proposed direct EPI-based pipeline outperforms the classical indirect T1w-based pipeline.

### 5.2. Residual inter-subject spatial variability

**Figure 4:**
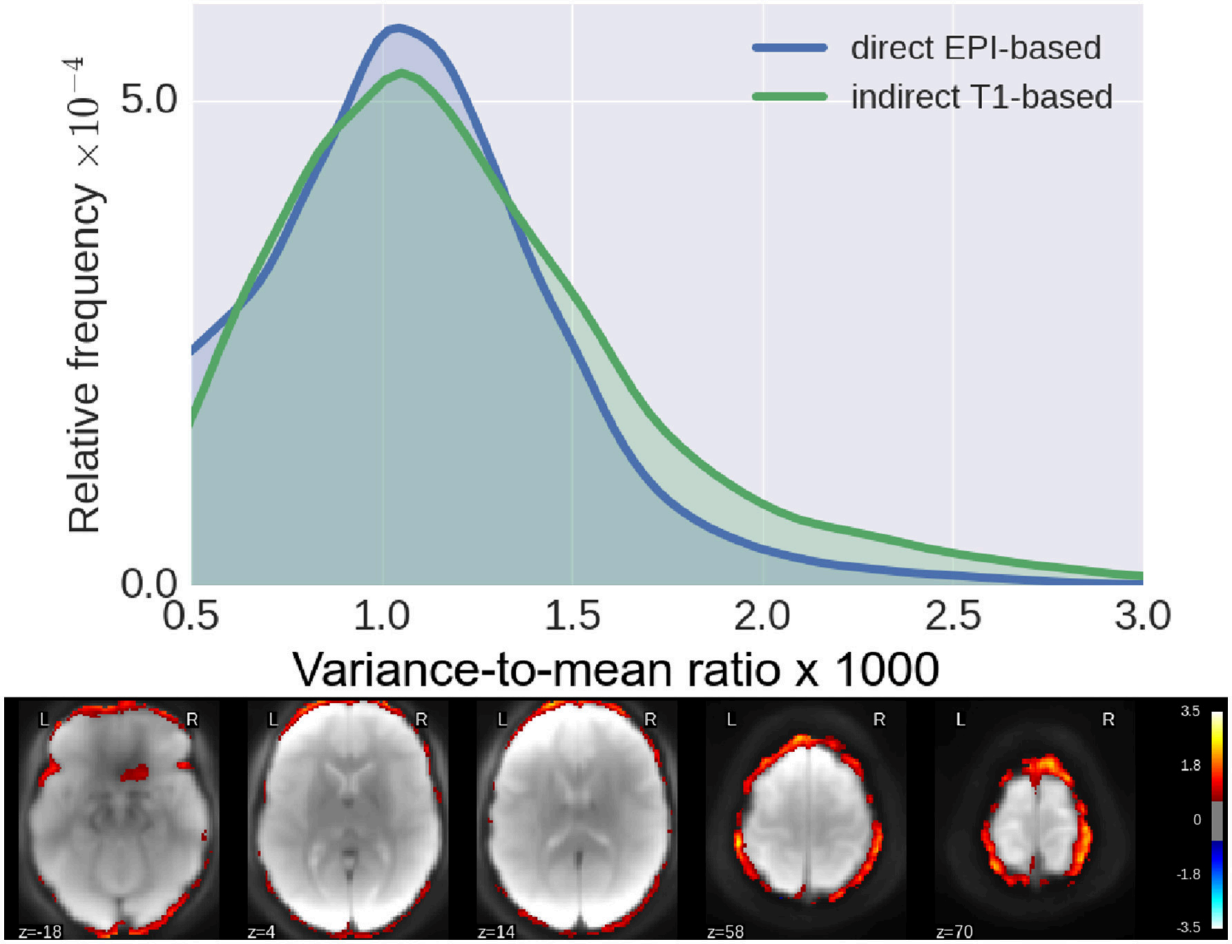
Residual inter-subject variability after registration. **(Top)** Histograms of relative variance [smaller is better, refer to Equation (4) for definition] for both pipelines. We see that our proposed method reduces the inter-subject variability by a much larger margin, indicative of improved subject-to-subject alignment. **(Bottom)** Log10 of ratio of the relative variance for indirect T1-based pipeline/direct EPI-based. Again, we see clearly that the gain of our proposed method is most pronounced along the cortical surface.

In Figure 4, we show histograms of across-subject per-voxel relative variance [refer to Equation (4) for formal definition]. We see that our proposed direct method outperforms the classical indirect T1w-based method, as the former leads to relatively more mis-aligned voxels across subjects, most concentrated on the outer edge of the cortex (see Figure 4).

### 5.3. Quality of estimated EPI group template

**Figure 5:**
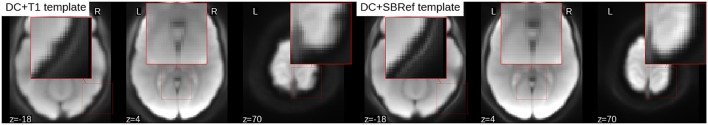
Mean EPI image across all subjects after registration (aka estimated population templates). Patches on the images have been zoomed to highlight details. The mean image from the indirect T1-based pipeline **(Left)** is more blurry (as seen here in the cerebellum), compared to our direct EPI-based pipeline post-registration across-subject mean image **(Right)** which is much sharper, indicative of a better inter-subject registration. Also the mean image from the T1-based pipeline has ripples on the cortical surface indicative of residual registration problems, that can be attributed to residual EPI-distortions not captured by coregistration.

To compare the quality of the group template produced by either pipeline, a snap hot of the resulting mean image or template is displayed in Figure 5. Compared to the proposed direct method, the mean image (across all subjects) from the indirect T1-based pipeline is blurry and has “ripples” on the cortical surface, indicative of residual mismatch between subjects after registration. The across-subject mean image post-registration with our direct EPI-based pipeline is the sharpest, showing that the subjects have been matched extremely well. Also, one notices that the mean image from the indirect T1-based pipeline still has some residual distortion (here in the left-to-right direction), even though distortion correction was done as part of both pipelines.

### 5.4. Group-level statistics maps and resting-state networks

**Figure 6:**
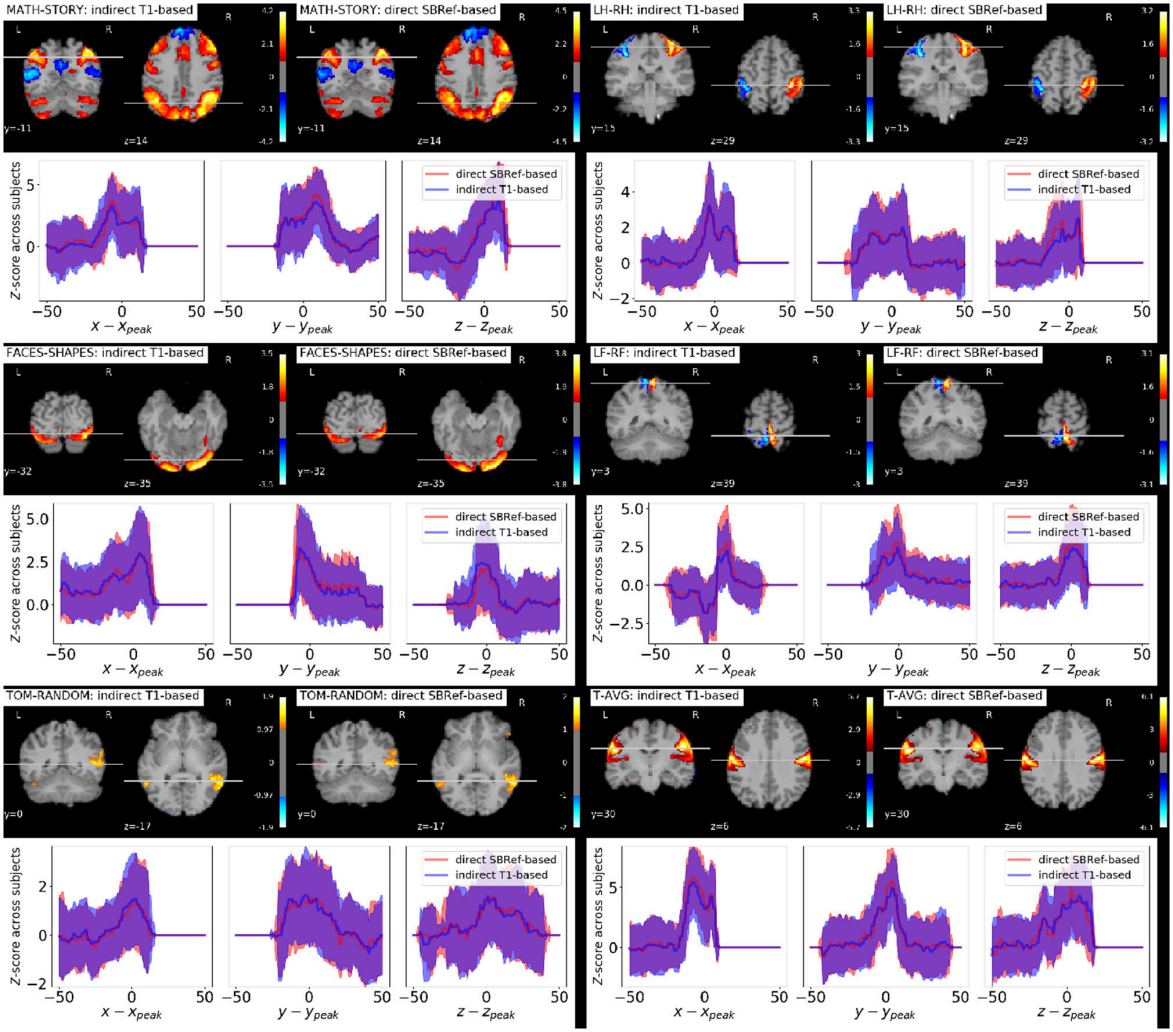
Qualitative comparison of pipelines via GLM results. For each task contrast, and for each registration pipeline (indicated in the legends), we plot across-subject mean activation maps of *Z*-scores. We also plot corresponding curves showing the variability of subject-specific activation *Z*-scores at brain locations within a 50 mm radius of *x, y*, and *z* coordinates of the group activation peaks. The figure reveals that the activation peaks across the different subjects are highly variable both in amplitude and spatial location. We see that our proposed direct EPI-based registration scheme leads to slightly higher activation peaks.

As regards group-level GLM scores, we see from Figure 6 that our proposed method performs as well as the classical indirect T1-based pipeline. This is remarkable, as the former pipeline does not use any anatomical data. However, as noted in Thirion et al. (2007) and Thyreau et al. (2012), the inter-subject variability in statistical maps results is not mainly due to misregistration, but to intrinsic subject differences that are manifested in amplitude differences: the response of subjects to the same stimulus/task is modulated differently, which is reflected in effect size fluctuations instead of position. This is confirmed in the curves in Figure 6, where we can see that the spatial across-subject activation profiles are very similar between the compared registration methods, except for the already noted slight improvement of the peak mean activation pattern obtained by our proposed method.

**Figure 7:**
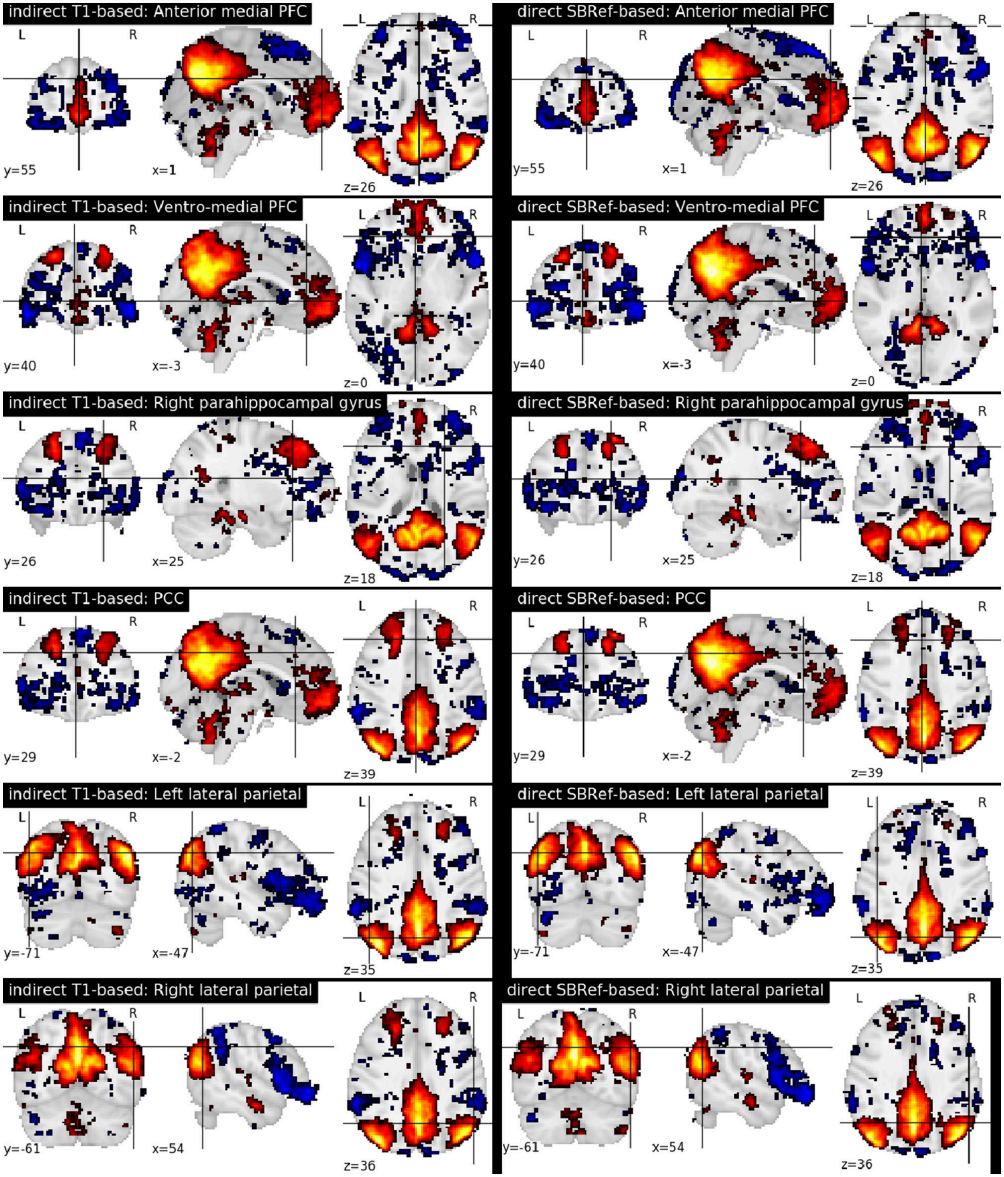
Comparing functional brain networks from subject fMRI images registered with both pipelines, namely the classical indirect T1-based method, and our proposed direct EPI-based method. Shown here are group-level unthresholded sub-component maps of the Default Mode Network (DMN) (Raichle et al., 2001), using MNI coordinates reported in Table 1 of Watanabe et al. (2013).

Finally, Figure 7 compares the functional brain networks obtained by running ICA on the images registered with each pipeline, and shows essentially the same network patterns. The absence of a difference between these maps can be explained by the fact that resting state networks are less focal than task-based activation-patterns, and so the former are less sensitive to the quality of the underlying registration procedure.

## 6. Discussion

Classical inter-subject registration pipelines use the T1 scan of a subject to estimate the subject-to-template warp. An obvious issue is that high-quality T1 scans are not always available, but more generally, it is not always possible to completely align the EPI images of a subject to their T1 image via coregistration. Added to this is the possibility that such an intermediate registration step is a potential source of interpolation artifacts. One should mention that surface-based methods are known to produce improved alignment of cortical landmarks over volume-based ones like the one produced here (e.g., Ghosh et al., 2010; Waehnert et al., 2016), but are orders of magnitude slower than volumetric methods, since the former involves intricate delineation of the geometry of cortical structures, and costly optimization over non-Cartesian grids (cortical meshes).

Further, as noted in Yamada et al. (2014), distortions cannot be fully removed with registration-based techniques, that are compensatory operations. Indeed, as shown by our experiments on the HCP dataset (Van Essen et al., 2012) (Figure 1), residual distortions persist even after correction. Consequently, efficient distortion correction for EPI data remains an open question. Our work proposes a direct EPI-based inter-subject registration pipeline that to some extent evades these bottlenecks.

We have proposed a computationally cheap EPI-based pipeline for direct inter-subject non-linear registration of functional data. Our method has been empirically validated on the HCP dataset (Van Essen et al., 2012), where we have shown that we obtain registered subject images with less inter-subject variability. Such direct EPI-based methods should replace the well-accepted classical T1-based strategy. Results on the HCP dataset (Van Essen et al., 2012) show that the proposed pipeline outperforms the classical T1-based indirect registration strategy, according to several quality metrics: NMI (Figure 3), residual inter-subject variance (Figure 4), and quality of estimated group template (Figure 5), without compromising the quality of post-registration statistical analyses results (GLM, ICA, etc.). These results replicate the findings of Grabner et al. (2014) and Huang et al. (2010) on a larger dataset with *n* = 110 subjects [compared to 10 subjects for Grabner et al. (2014) and 16 subjects for Huang et al. (2010)] at a much lower resolution of 2 mm isotropic [compared to 1.1 mm isotropic in Grabner et al. (2014) and 1 mm isotropic in Huang et al. (2010)]. Still using an EPI-based strategy, the work (Calhoun et al., 2017) has shown similar results as ours on the ABIDE dataset (Di Martino et al., 2014).

Our experiments show that according to low-level QA metrics like NMI (Figure 3), residual inter-subject spatial variability (Figure 4), and the quality of across-subject mean registered EPI image (Figure 5), our proposed method outperforms the classical indirect T1-based registration. It is well known that volume-based registration strategies fail to match precisely many cortical areas. Surface-based methods (Hinds et al., 2008; Tucholka et al., 2012; Renvall et al., 2016) partly solve this problem and could potentially help reduce the variance for indirect T1w-based pipeline, yet with a potential increase in computation time.

In terms of more high-level metrics like group-level GLM statistics, these gains are still present, though not as pronounced (refer to Figure 6). Indeed, as noted in Thirion et al. (2007), Thyreau et al. (2012), and Xu et al. (2009) the inter-subject variability in brain maps is not primarily due to misregistration, but to between subject differences in the magnitude and precise shape of activation patterns. They will not be reduced by improved anatomical alignment. Indeed Tavor et al. (2016) showed that resting-state fMRI data alone (no anatomical features like brain tissue maps, etc.) can be used to predict the activation maps of a subject to a task, well above chance. This proved, amongst other things, that task-based brain activations are largely physiological—in contrast to being driven by subjects' brain morphological differences—and can be predicted from resting state fMRI data.

In a separate work (Dohmatob et al., 2016), we have considered the possibility to model explicitly this physiology differences by estimating latent factors of variability across-subjects in a data-driven way using dictionary-learning. The motivating idea behind such a model, is that activation would be governed by the same generative model (the latent model), and modulated at the individual level by subject-specific physiology.

## Author contributions

ED: Main contributor, all aspects of the work (conception, methods, experiments, writing, etc.); GV: Ph.D. co-advisor, contributed to all aspects of the work; BT: Ph.D. advisor, contributed to all aspects of the work.

### Conflict of interest statement

The authors declare that the research was conducted in the absence of any commercial or financial relationships that could be construed as a potential conflict of interest.

## Footnotes

^1^*Pyramidal* means that multiple passes are made by a registration algorithm on the input images, with finer and finer resolution (aka *pyramid*). In this speedup technique, each pass of the pyramid is initialized with the solution of the previous pass (this is known as *warmstarting*).

^2^The “°” symbol denotes composition of transformations.

^3^With the “*-schedule*” option.
